# A Promising Prognostic Signature Consisting of Fatty Acid Metabolism Genes based on Machine Learning Predicts Biochemical Recurrence and Aids ARSI Therapy in Prostate Cancer

**DOI:** 10.7150/jca.112597

**Published:** 2025-07-28

**Authors:** Zhenjie Wu, Yulin Deng, Yuanfa Feng, Zeheng Tan, Shanghua Cai, Huichan He, Wenjun Yin, Jinchuang Li, Weicheng Tian, Jianming Lu, Wenjie Xie, Jundong Lin, Jianheng Ye, Zhaodong Han, Weide Zhong

**Affiliations:** 1 Department of Urology, The Second Affiliated Hospital, School of Medicine, South China University of Technology, Guangzhou, 510180, People's Republic of China.; 2 Department of Urology, The First Dongguan Affiliated Hospital, Guangdong Medical University, Dongguan, 523710, People's Republic of China.; 3 Guangdong Provincial Key Laboratory of Urology, Department of Urology, The First Affiliated Hospital of Guangzhou Medical University, Guangzhou Medical University, Guangzhou, 510120, People's Republic of China.; 4 Guangzhou National Laboratory, No. 9 XingDaoHuanBei Road, Guangzhou International Bio Island, Guangzhou, 510005, People's Republic of China.; 5 State Key Laboratory of Quality Research in Chinese Medicine, Macau University of Science and Technology, Taipa, 999078 Macau, People's Republic of China.

**Keywords:** prostate cancer, fatty acid metabolism, biochemical recurrence, risk stratification, ARSI therapy

## Abstract

**Background:** Fatty acid metabolism (FAM) is a crucial metabolic characteristic of tumor cells, playing a role in various pathological processes during tumor development. Till now, the prognostic role of FAM-related genes of prostate cancer (PCa) is far from fully investigation.

**Methods:** The combinations of 10 machine learning algorithms were applied in this study. A reliable signature, FAM-related gene score (FAMRGs), was developed to predict the prognosis of patients with PCa. External data sets were used to verify the accuracy and robustness of the FAMRGs. Drug sensitivity analysis was used to predict the optimal drug for high-risk PCa patients. The underlying mechanism related to FAMRGs were investigated by functional enrichment analysis. A nomogram based on FAMRGs was developed for personalized prediction of patient prognosis.

**Results:** A stable FAMRGs was construced and validated in 6 independent cohorts. FAMRGs accurately divided PCa patients into low and high risk group. FAMRGs showed stronger predictive ability compared with published prognostic signatures for PCa. Also, the androgen receptor signaling inhibitors (ARSI) treatment response predictive ability of FAMRGs was identified. Five drugs that were most suitable for patients in the high risk group of FAMRGs were screened. It was shown that FAMRGs involved in cell cycle-related pathways. The novel nomogram showed precisely predictive ability for the outcomes of patients with PCa.

**Conclusions:** The FAMRGs can accurately predict the prognosis of PCa patients and is expected to direct the clinical treatment for PCa.

## 1. Introduction

Prostate cancer (PCa) is the second most commonly diagnosed cancer and a leading cause of cancer-related mortality in men globally. In 2024, it is reported that there will be 299,010 new cases of PCa in the United States, representing 29% of all new male cancer cases, and an estimated 35,250 deaths, which would account for 11% of male cancer deaths 1. The primary therapeutic approaches for PCa include surgery, radiotherapy, chemotherapy and endocrine therapy. However, these treatments have certain side effects and limitations 2-4. Even treated with appropriate therapy, an increased risk of biochemical recurrence (BCR) remains insidious for high-risk PCa patients, leading to a lethal stage of the disease 5, 6. At present, adjuvant treatments have been applied in clinical practice to improve the prognosis of PCa patients 7, 8. Unfortunately, due to the limitations of existing testing methods, accurately predicting BCR of high-risk PCa patients is emergently needed 9.

Currently, the detection of BCR mainly relies on the monitoring of serum prostate specific antigen (PSA) 10. After a radical prostatectomy (RP), abnormally elevated PSA level may indicate a recurrence or residual tumor 5, 11. However, elevated PSA levels cannot be ruled out as being caused by non-cancerous prostate disease, and relying solely on PSA level testing to assess the prognosis of PCa patients may lead to overdiagnosis and overtreatment 12. In recent years, the utilization of tumor next-generation sequencing (NGS) technology within the realm of oncology has been expanding 13. Various prognostic models based on gene expression have gradually become important tools to guide the personalized treatment of PCa and other cancers. However, the majority of published models face challenges in clinical implementation due to poor selection of algorithms for building models. Hence, the development of a prognostic model utilizing machine learning method still has considerable room for enhancement in clinical application.

Fatty acid metabolism (FAM), as an important component of lipid metabolism, is involved in various cell biological processes, such as cell proliferation, energy conversion and organelle synthesis 14, 15, promoting the pathological processes of tumor formation 16. Abnormally activated lipid metabolism enables most tumor cells to proliferate through synthesis, elongation and desaturation of fatty acids (FAs), leading to tumor growth, invasion and metastasis 17. CD147 was found to promote fatty acid synthesis and inhibit fatty acid beta-oxidation, thus enhancing the proliferation and metastasis of hepatocellular carcinoma cells 18.

Recently, most studied focused on the molecular mechanisms of FAM among the progression of malignant tumors. Additionally, prognostic models constructed based on FAM-related genes showed potential clinical value for the treatment responses of patients with advanced rectal cancer 19 and glioma 20. Nevertheless, whether FAM-related genes can serve as predictive factors for PCa remains far from fully investigated.

In this study, we collected and collated 6 PCa clinical cohorts, based on differentially expressed FAM-related genes in PCa, tempting to construct a novel prognostic signature applying comprehensive machine learning strategy to identify PCa patients at high risk of BCR. We verified the feasibility of the signature in the independent cohorts and predicted the most appropriate agents for high-risk BCR patients, hoping to help guide the individualized treatment of PCa.

## 2. Materials and methods

### 2.1 Data sources

The detailed information of 7 PCa public datasets applied in this study were shown in Supplementary Table s1. The RNA-sequencing data and corresponding clinical data of PCa patients were obtained from the Cancer Genome Atlas (TCGA, https://cancergenome.nih.gov/), the cBioPortal for Cancer Genomics (https://www.cbioportal.org/), and Gene Expression Omnibus (GEO, https://www.ncbi.nlm.nih.gov/geo/). Trimmed Mean of M-values (TMN) method in edgeR package was applied to process the RNA-seq data of TCGA. Robust Multichip Average (RMA) method in oligo package was used to process the Affymetrix microarray data. The gene expression values were standardized through log2 transformation.

### 2.2 Identification of BCR-related FAM candidate genes

Genes in fatty acid metabolism pathways were acquired from Molecular Signatures Database (MSigDB, https://www.gsea-msigdb.org/gsea/msigdb). The limma package was used to identify differentially expressed genes (DEGs) between PCa and normal tissue in TCGA with thresholds of |log2 fold change (FC)| > 1 and adjusted P < 0.05. Univariate Cox regression analysis (P < 0.05) was employed to identify FAM BCR-related genes.

### 2.3 Construction of a prognostic FAM-related gene score (FAMRGs) for patients with PCa

To construct a reliable FAMRGs accurately forecasting the outcomes of PCa patients with stable performance, 117 combinations of 10 machine learning algorithms were intergrated. The 10 machine learning algorithms employed in this study included CoxBoost, elastic network (Enet), generalized boosted regression modeling (GBM), partial least squares regression for Cox (plsRcox), random survival forest (RSF), Ridge, stepwise Cox, supervised principal components (SuperPC), survival support vector machine (suvival-SVM), and the least absolute shrinkage and selection operator (Lasso). We identified the optimal algorithm combination based on the highest average C-index and the best predictive performance.

### 2.4 The expression feature and prognostic role of genes in FAMRGs

The mRNA expression levels of FAMRGs in normal prostate tissues and PCa tissues were obtained from TCGA and compared by Student's t test. The protein expression of FAMRGs in normal prostate and PCa tissues were assessed by applying the Human Protein Atlas (HPA) database (https://www.proteinatlas.org/) under the citation guidelines of HPA. The URLs that link directly to the detailed information of the images on the site were displayed in the Supplementary Table s2. Univariate Cox regression and multivariate Cox regression were applied to assess the prognostic value of genes in FAMRGs.

### 2.5 Evaluation of the FAMRGs across 6 independent cohorts

Kaplan-Meier (K-M) survival curves of BCR were performed by survival package. The receiver opearting characteristic (ROC) curves were performed by survivalROC package. The survminer package was applied to determine the optimal cut-off value with the parameter 'minprop' = 0.1. Patients were stratified into high- and low- risk groups based on this cut-off for K-M analysis. Univariate Cox regression analysis was applied to evaluate the prognostic value of the signature.

### 2.6 Comparison of the predictive performance of FAMRGs and published signatures

To further validate the predictive performance of FAMRGs, we conducted a systematic search on PubMed and collected 51 prognostic signatures of PCa. These signatures were linked to multiple biological processes including lipid metabolism, glycolysis, apoptosis, ferroptosis, hypoxia, and inflammation. C-index of each prognostic signature in 6 cohorts were calculated for comparison.

### 2.7 Androgen receptor signaling inhibitors (ARSI) treatment response predictive ability of FAMRGs

ARSI cohort was downloaded from the cBioPortal database. Specifically, 75 ARSI treatment samples with gene expression and prognostic data were acquired from the PRAD SU2C 2019 dataset.

### 2.8 Calculating the potential sensitive therapeutic drugs for patients with high FAMRGs

Drug sensitivity data were downloaded from the Cancer Therapeutics Response Portal (CTRP, https://portals.broadinstitute.org/ctrp.v2.1/). The IC50 values of different drugs for each sample in TCGA were assessed by calcPhenotype function. Pearson correlation analysis was applied to perform the correlation between risk scores and IC50 values.

### 2.9 Enrichment analysis

We downloaded h.all.v2024.1.Hs.symbols.gmt and c2.cp.kegg_legacy.v2024.1.Hs.symbols.gmt from Molecular Signatures Database (MSigDB, https://www.gsea-msigdb.org/gsea/msigdb). DEGs between the high- and low- risk groups of FAMRGs were identified using the R package limma and ranked in descending order according to the log2 fold change (log2FC). The ranked gene list was then further analyzed using the R package clusterProfiler for gene set enrichment analysis (GSEA) to identify significant enriched functional pathways. Gene ontology (GO) enrichment analysis was employed to investigate the major biological process. Gene sets with a q-value < 0.05 were considered significantly enriched.

### 2.10 Establishment of a novel nomogram

A novel nomogram for BCR, consisting of pathological T (pT) stage, pathological N (pN) stage, clinical M (cM) stage, age, gleason score, PSA value, and risk score of FAMRGs, was constructed by rms package. Decision curve analysis was performed to assess the clinical benefit of prediction models. ROC curves were applied to assess the predictive ability of the nomogram. The web-based BCR probability calculators were built using R packages DynNom and shiny.

### 2.11 Statistical analysis

All statistical analysis and visualizations were performed using R software (version 4.1.0). The survminer package was applied to determine the optimal cut-off value. Statistical significance was considered at P < 0.05.

## 3. Results

### 3.1 Construction of a prognostic signature consisting of 13 FAM genes (FAMRGs) predicting BCR of PCa

**Figure 1:**
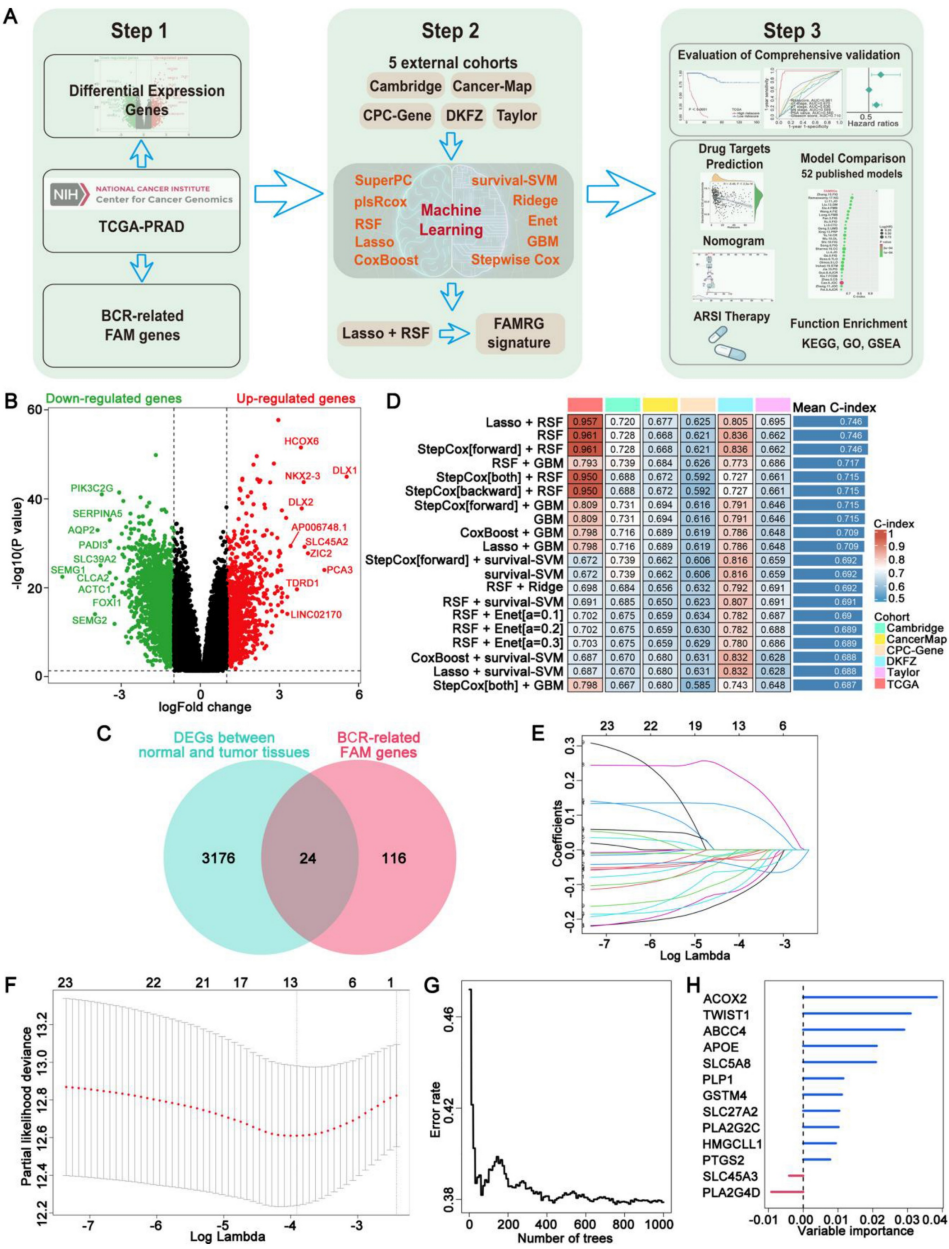
** Construction of a signature consisting of 13 FAM genes (FAMRGs) predicting BCR of PCa. (A)** Flow chart of this study. **(B)** The volcano plot indicating the DEGs between normal and tumor tissues. Thresholds include a P value < 0.05 and ïlog2FCï > 1. **(C)** The venn diagram of DEGs between normal and tumor tissues and BCR-related FAM genes. **(D)** The C-indexes of the top 20 of 117 machine-learning algorithm combinations in the six cohorts. **(E)** The coefficients in Lasso regression analysis. **(F)** Selection of lambda in the Lasso regression model. The error rate curve of the RSF algorithm **(G)** and the variable importance of the 13 core genes **(H)**.

This study was carried out in adherence to the workflow charted in Figure 1A. 3,200 DEGs were identified between normal and Pca tissue in TCGA with *P* < 0.05 and |log2FC| > 1 (Figure 1B). Then, univariate Cox regression analysis was performed, utilizing a p-value threshold of 0.05, to identify 140 BCR-related FAM genes. Venn plot revealed that a total of 24 marker genes were intersected between DEGs and BCR-related FAM genes, and these genes served as the basis for the subsequent analysis (Figure 1C). Then, a integration approach based on machine learning was employed to develop an accurate and reliable FAMRGs. Using 10 different machine learning algorithms, we integrated 117 types of prediction models (Supplementary Table s3). The C-index for each model in the TCGA training cohort and 5 external validation cohorts (Cambridge, CancerMap, CPC-Gene, DKFZ, and Taylor) were calculated and ranked. The top 20 combinations were presented in Figure 1D, the combination of Lasso and RSF algorithms exhibited the highest average C-index of 0.746 and was selected as the optimal model. In the Lasso regression, the optimal lambda was identified when the partial likelihood of deviance reached the minimum value, resulting in the selection of 13 genes of utmost value (Figures 1E and F). These genes were subsequently optimized using the RSF algorithm, which improved the performance of the model and led to the enhancement of a highly robust prognostic model named FAMRGs. The prediction error rate remained low and stable when constructing 1000 survival trees (Figure 1G). The variable importance (VIMP) of each gene indicated its contribution to the prediction of BCR. Finally, a gene set of 13 core genes (ACOX2, TWIST1, ABCC4, APOE, SLC5A8, PLP1, GSTM4, SLC27A2, PLA2G2C, HMGCLL1, PTGS2, SLC45A3, and PLA2G4D) was identified (Figure 1H) and predict function was applied to score each sample.

Compared with mRNA expression in normal prostate tissue, SLC27A2, TWSIT1, SLC45A3, APOE and ABCC4 were upregulated, while ACOX2, GSTM4, PLA2G2C, PLP1, PTGS2, HMGCLL1, PLA2G4D and SLC5A8 were downregulated in PCa tissue (Figure s1A). Immunohistochemical information from the HPA database showed that higher staining of GSTM4, PLP1, and PTGS2 was found in normal prostate tissue, while higher staining of SLC27A2, SLC45A3, APOE, and ABCC4 was observed in prostate cancer tissue (Figure s1B). Futhermore, univariate Cox regression analysis found that all genes in FAMRGs were prognostic factors in PCa (Figure s1C), and two genes (TWIST1 and APOE) were indentified as independent prognostic factors by multivariate Cox regression analysis (Figure s1D).

### 3.2 The prognostic significance of FAMRGs

**Figure 2:**
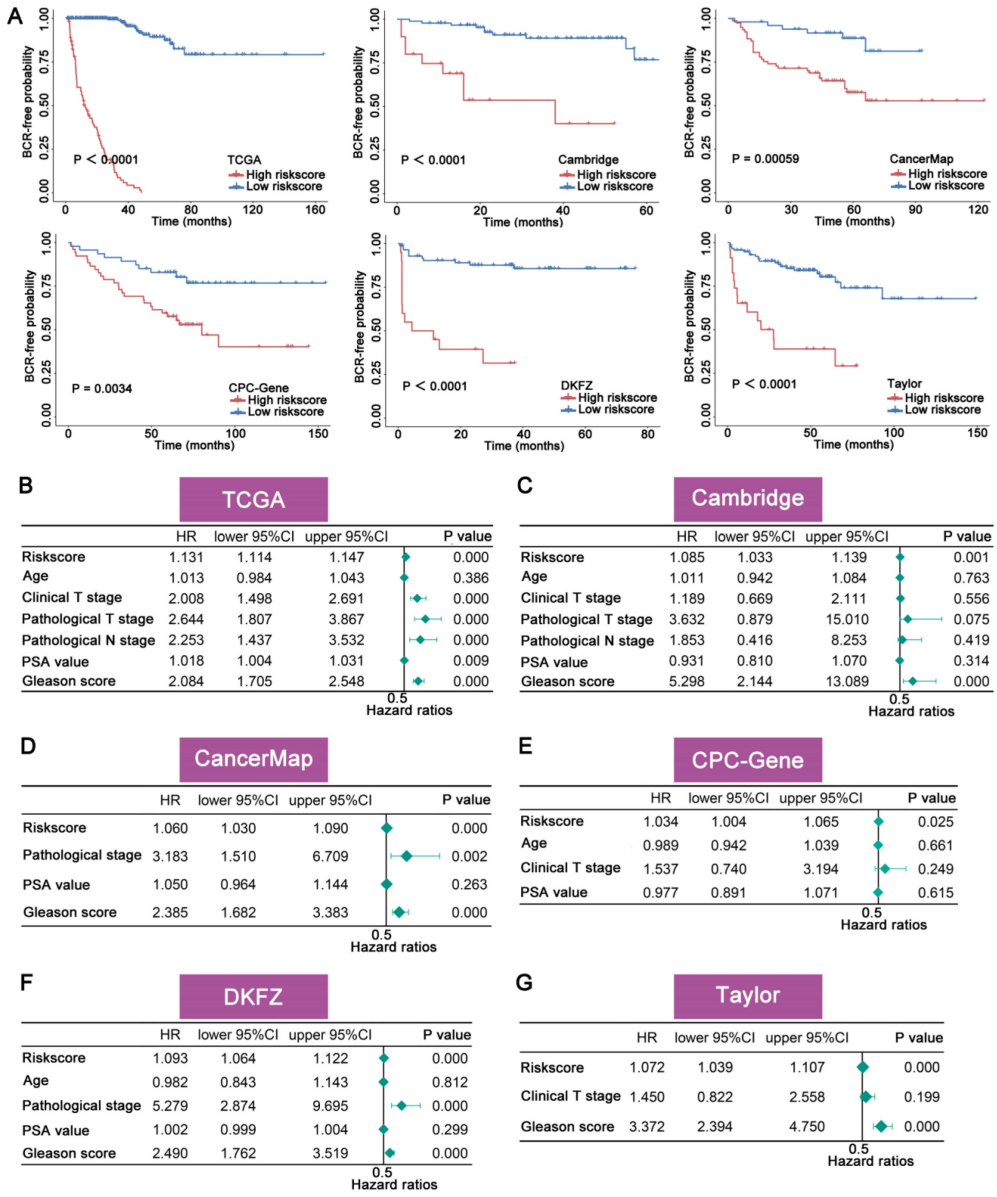
** The prognostic significance of FAMRGs. (A)** The K-M survival curves for the patients from TCGA, Cambridge, CancerMap, CPC-Gene, DKFZ and Taylor cohorts. **(B-G)** Univariate Cox regression analysis of riskscore and pathological clinical characteristics of the six cohorts, respectively.

To assess the prognostic significance of the FAMRGs, we categorized the samples within each cohort into high and low risk groups using the optimal cutoff value. K-M survival analysis revealed a significant difference in BCR rates between high and low risk groups in the training cohort TCGA (N = 463, P < 0.0001) and the validation cohorts, including Cambridge (N = 111, P < 0.0001), CancerMap (N = 127, P = 0.00059), CPC-Gene (N = 99, P = 0.0034), DKFZ (N = 105, P<0.0001), and Taylor (N = 140, P < 0.0001), indicating that patients in the high risk group were more likely to develop BCR (Figure 2A).

Univariate Cox regression analysis (Figures 2B-G) showed that FAMRGs could act as a predictive indicator for BCR in TCGA (HR = 1.131, P = 0.000), Cambridge (HR = 1.085, P = 0.001), CancerMap (HR = 1.060, P = 0.000), CPC-Gene (HR = 1.034, P = 0.025), DKFZ (HR = 1.093, P = 0.000), and Taylor (HR = 1.072, P = 0.000). In contrast to clinical factors like age, PSA, Gleason score and TMN, FAMRGs was identified as a reliable independent prognostic factor for BCR in all cohorts.

### 3.3 Evaluation of the accuracy and robustness of FAMRGs

**Figure 3:**
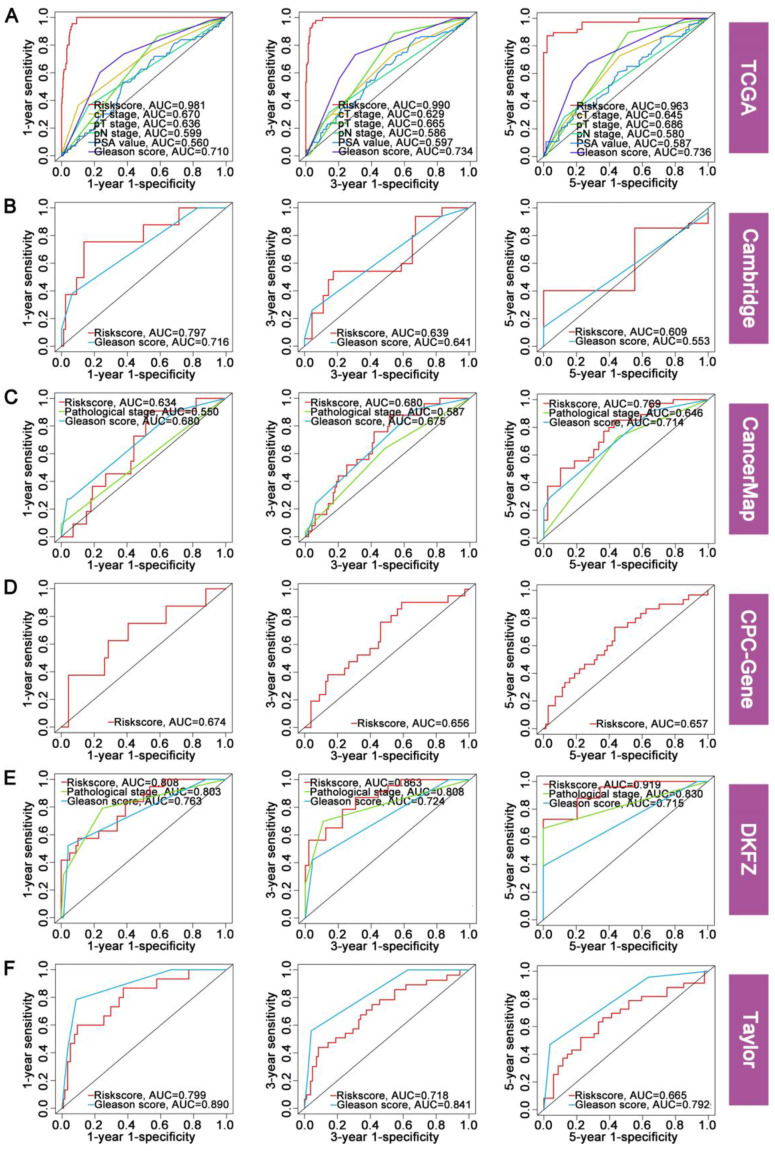
** Evaluation of the accuracy and robustness of FAMRGs. (A-F)** The ROC curves of 1-year, 3-year and 5-year BCR prediction for the TCGA, Cambridge, CancerMap, CPC-Gene, DKFZ and Taylor cohorts, respectively.

The accuracy and robustness of FAMRGs were further confirmed through ROC analysis. The AUC values in each cohort were used to evaluate the predictive accuracy of FAMRGs and clinical factor. The 1-, 3-, and 5-year AUC values of FAMRGs were 0.981, 0.990, and 0.963 in TCGA (Figure 3A); 0.797, 0.639, and 0.609 in Cambridge (Figure 3B); 0.634, 0.680, and 0.769 in CancerMap (Figure 3C); 0.674, 0.656, and 0.657 in CPC-Gene (Figure 3D); 0.808, 0.863, and 0.919 in DKFZ (Figure 3E); and 0.799, 0.718, and 0.665 in Taylor (Figure 3F), respectively. Overall, the AUC values of FAMRGs had better performance than clinical characteristics in most cohorts, except Taylor, indicating the accurate and robust predictive performance of FAMRGs.

### 3.4 Comparison of predictive significance between FAMRGs and 51 published signatures

**Figure 4:**
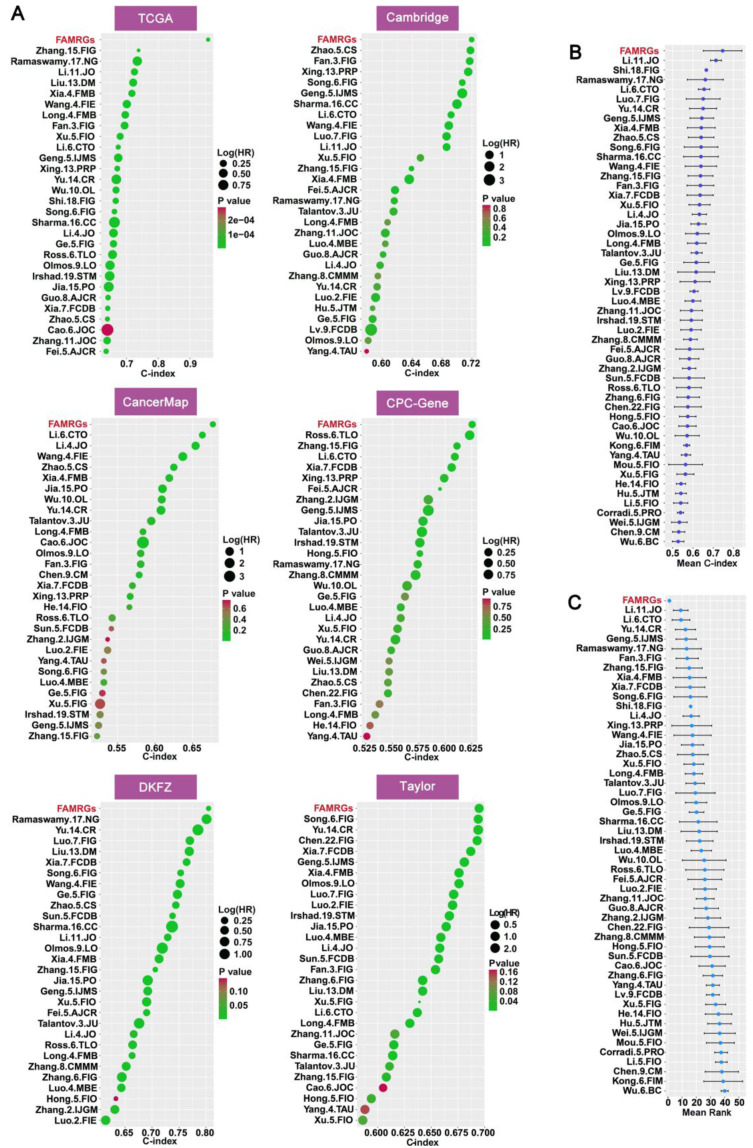
** Comparison of prognostic value between FAMRGs and 51 published signatures. (A)** The top 30 C-indexes of FAMRGs and 51 published signatures are presented in TCGA, Cambridge, CancerMap, CPC-Gene, DKFZ and Taylor cohorts. **(B)** The average C-index of FAMRGs and 51 published signatures in the six cohorts. **(C)** The average rank of FAMRGs and 51 published signatures in the six cohorts. The error bars indicate the 95% confidence interval (CI).

To further validate the prognostic value of FAMRGs in comparison to various published predictive signatures for PCa, we complied 51 published signatures associated with diverse biological characteristics, including lipid metabolism, glycolysis, cell apoptosis, ferroptosis, hypoxia, and inflammation. We then computed the C-index for the 51 signatures across the six cohorts. In the comparison of C-index among the 51 signatures and FAMRGs, the top 30 of each cohort were shown in Figure 4A. In addition, FAMRGs significantly outperformed other signatures in the overall average C-index (Figure 4B) and ranking (Figure 4C). These results suggested that FAMRGs exhibited robust predictive and generalization capabilities when compared with other signatures.

### 3.5 The performance of FAMRGs in predicting the efficacy of ARSI treatment for patients with PCa

**Figure 5:**
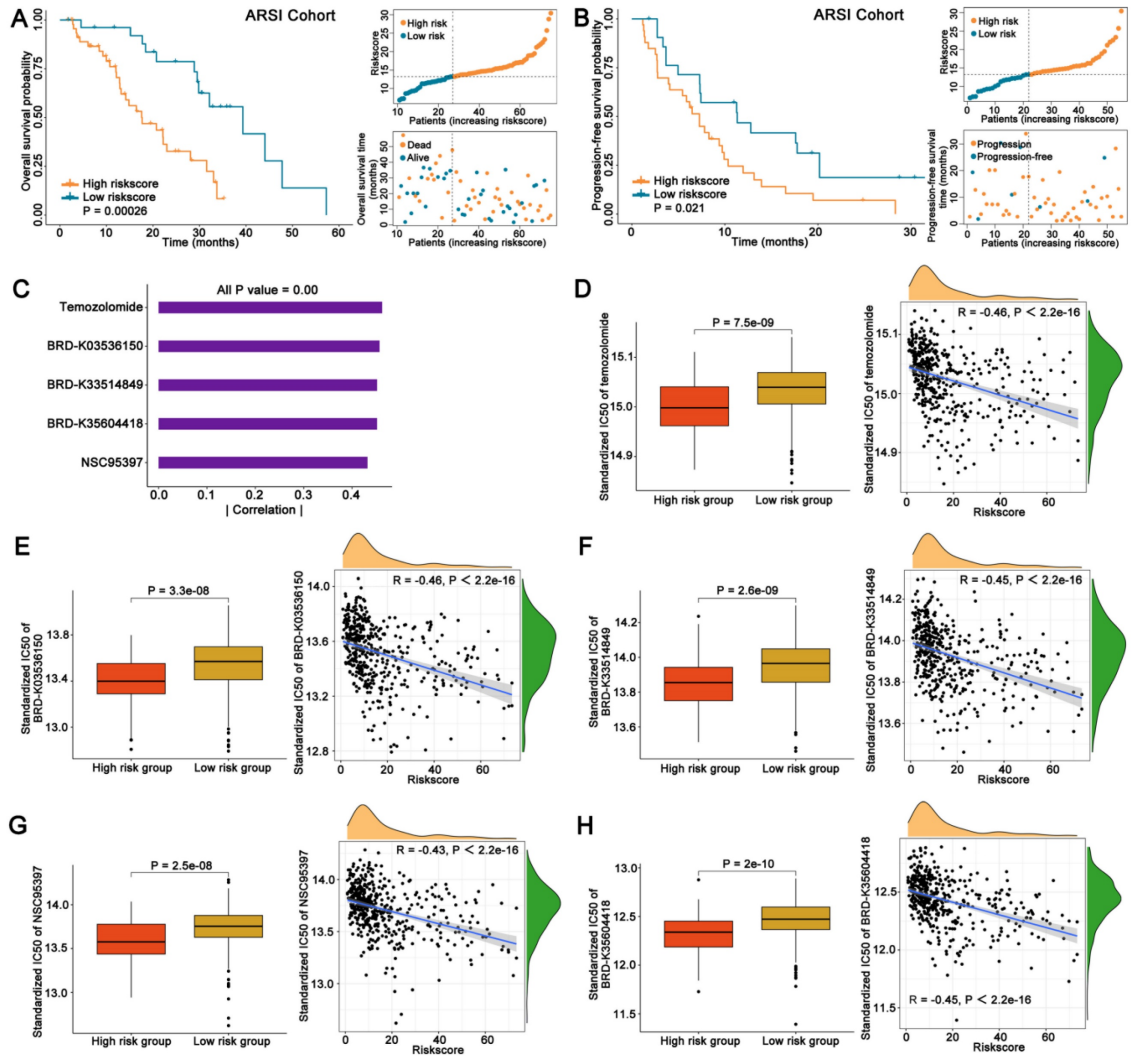
** FAMRGs in ARSI and sensitivity therapeutic agents prediction. (A)** The K-M survival analysis of overall survival for patients from ARSI cohort. **(B)** The K-M survival analysis of progression-free survival for patients from ARSI cohort. **(C-H)** Potential pharmaceutical compounds are presented in the high FAMRGs group.

To better evaluate the clinical value of FAMRGs, we observed the ability of the FAMRGs in forecasting the response to ARSI therapy. Patients with low FAMRGs presented a more favorable overall survival and progression-free survival than those with high FAMRGs (Figures 5A and B).

### 3.6 Identification of potential therapeutic agents for patients with high FAMRGs

Drug response data were obtained from the CTRP database and Spearman correlation analysis (|Spearman's R| > 0.4) was employed to identify the top five agents exhibiting a considerable negative correlation between IC50 and FAMRGs (Figure 5C). The IC50 values and their correlations with FAMRGs for the five agents derived from CTRP, including Temozolomide, BRD-K03536150, BRD-K33514849, BRD-K35604418 and NSC95397 were showed in Figures 5D-H, respectively. These five agents all exhibited lower IC50 values in high FAMRGs group and demonstrated a negative correlation with FAMRGs, potentially offering therapeutic alternatives for patients with high FAMRGs.

### 3.7 The biological function related to FAMRGs

**Figure 6:**
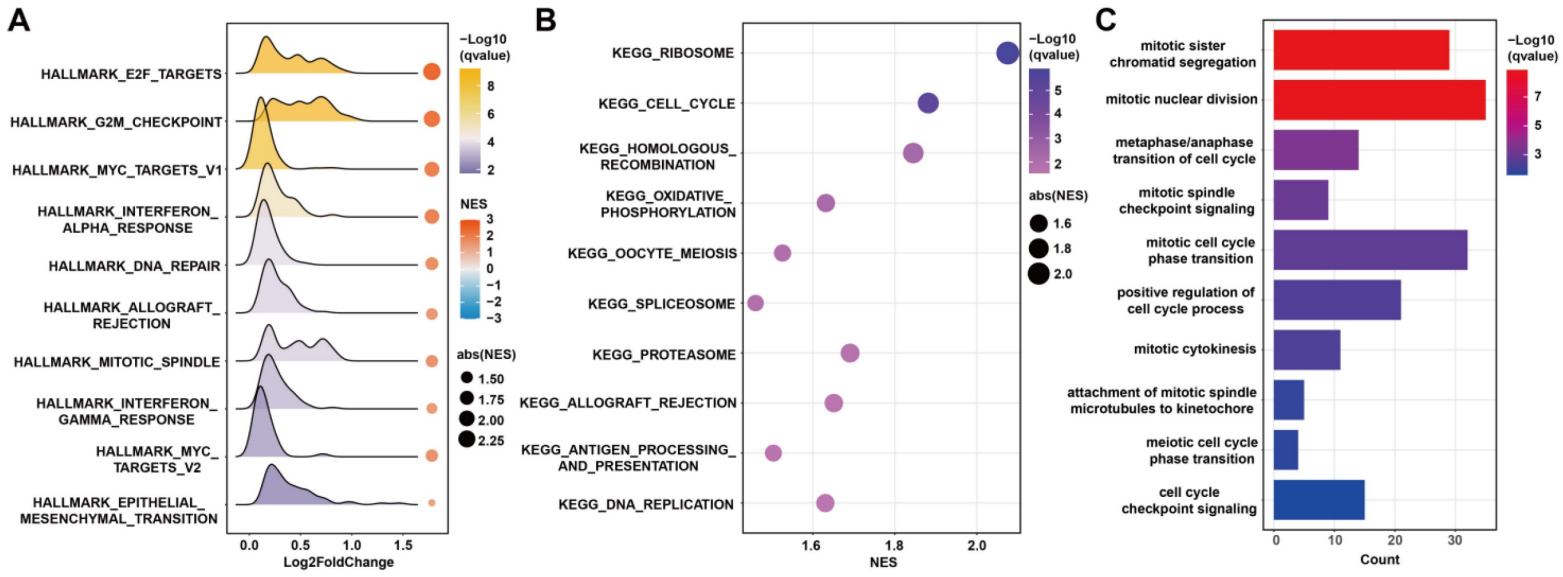
** Functional enrichment analysis of FAMRGs. (A-B)** Gene set enrichment analysis (GSEA) of FAMRGs. **(C)** Gene ontology (GO) enrichment analysis of FAMRGs.

To explore the biological functions related to FAMRGs, GSEA analysis was conducted and found that FAMRGs was enriched in pathways related to tumor growth, such as E2F targets, G2M checkpoint, mitotic spindle, cell cycle, and DNA replication (Figures 6A and B). The GO analysis revealed that the most related biological processes of FAMRGs were mitotic sister chromatid segregation and mitotic nuclear division (Figure 6C).

### 3.8 Construction of a novel nomogram consisted of FAMRGs for clinical utilization

**Figure 7:**
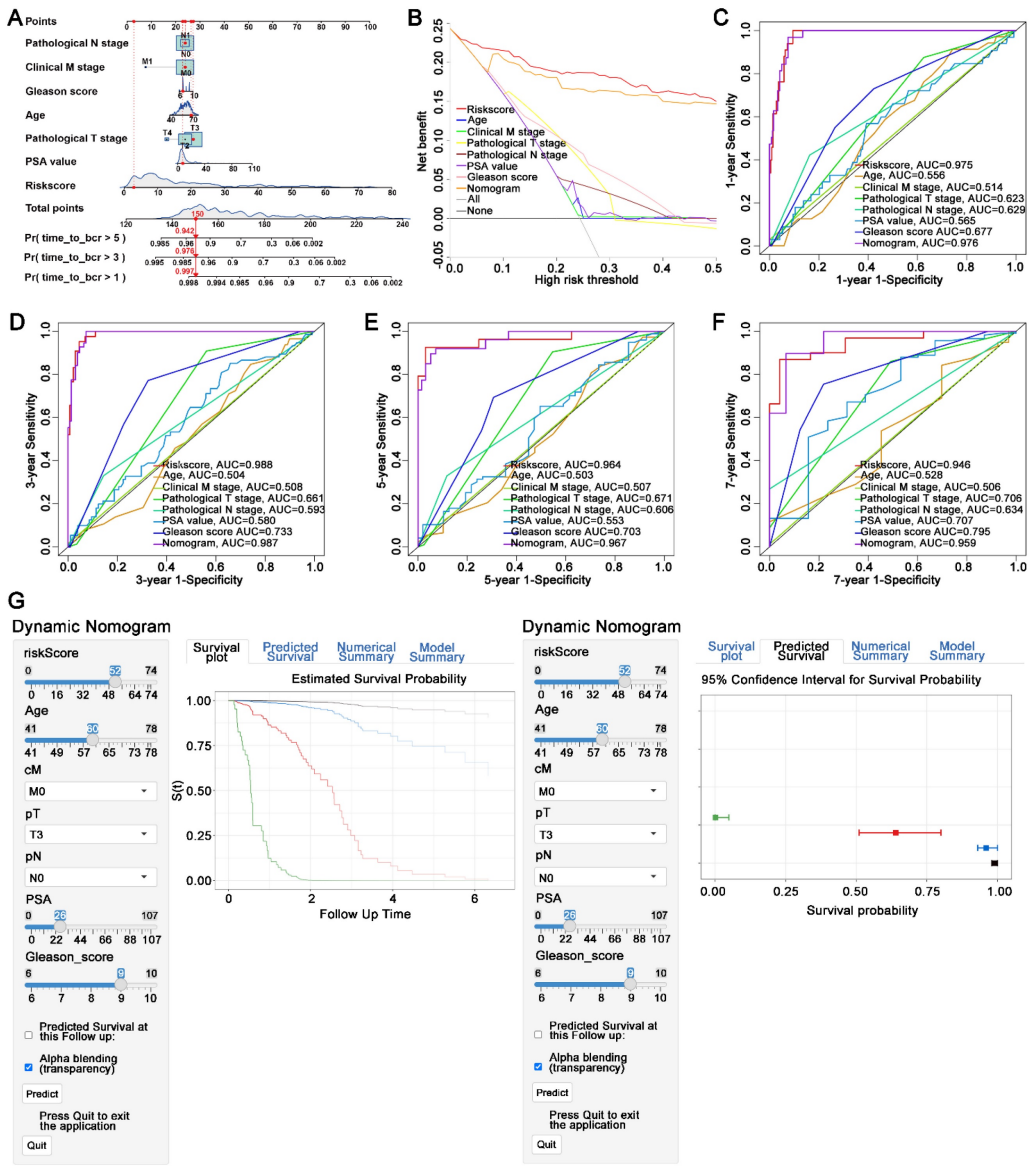
** Construction of a novel nomogram consisting of FAMRGs for predicting survival. (A)** Nomogram integrates FAMRGs and clinicopathological characteristics. **(B)** Decision curve analysis of the nomogram and clinicopathological characteristics for survival prediction. **(C-F)** The 1-year, 3-year, 5-year, and 7-year survival ROC curves of the nomogram, FAMRGs, and clinicopathological characteristics. **(G)** A dynamic nomogram integrates FAMRGs and clinicopathological characteristics for clinical application to predict survival. The K-M analysis is shown on the left. The corresponding 95% CI is shown on the right.

A novel nomogram was established including the pT stage, pN stage, cM stage, gleason score, age, PSA value, and risk score of FAMRGs. The risk score was identified as the primary contributor to BCR prediction for PCa patients in the novel developed nomogram (Figure 7A). Decision curve analysis demonstrated that the nomogram had a greater clinical net benefit than clinical characteristics, except for the risk score (Figure 7B). Furthermore, the 1-, 3-, 5-, and 7-year AUC values of the nomogram for predicting BCR were 0.976, 0.987, 0.967, and 0.959, respectively (Figures 7C-F). To enhance the clinical application of the novel nomogram, we developed a dynamic nomogram (Figure 7G) that allowed for more convenient and intuitive prediction of BCR probability according to the individual characteristics of PCa patients (https://410studio.shinyapps.io/DynNomapp/).

## 4. Discussion

BCR is recognized as a risk factor for possible clinical metastasis and unfavorable prognosis in PCa patients 21. Early detection and prompt intervention can significantly improve the prognosis of PCa patients 22. Currently, the precision in distinguishing high risk patients through measures like PSA levels, Gleason score, tumor invasion, and metastatic status is inadequate 12. Therefore, it is crucial to discover novel prognostic biomarkers and risk scores that can accurately stratify the risk of BCR in patients with PCa.

FAM is a significant metabolic characteristic of tumor cells and participates in various pathological mechanisms. PCa is rich in fatty acids, and the abnormal regulation of FAM promotes the poor prognosis 23. In previous studies, FAM-related genes were used to construct prognostic models of PCa 24. Due to the deficiencies in the selection of algorithms and the number of validation cohorts, the predictive accuracy of the model requires enhancement. Here, we collected multiple cohorts from public databases and applied machine learning to select the most suitable algorithm combination to construct a novel prognostic signature. The combination of Lasso and RSF algorithms yielded the highest C-index and was used to construct the FAMRGs, which contained 13 core genes. Also, FAMRGs performed better accuracy and robustness, compared with various preditive models of PCa. To the best of our knowledge, FAMRGs is the first predictive model constructed by comprehensive machine learning algorithm combination and obtaining a high level of testing efficiency.

Seven genes in FAMRGs have been reported to be associated with the progression of PCa. We have demonstrated that ACOX2 inhibits PCa progression by regulating fatty acid oxidation 25. TWIST1 was found to direct an embryonic developmental program for prostate organogenesis, thereby facilitating cancer metastasis 26. ABCC4 was shown to play a key role in docetaxel resistance in PCa 27. APOE can promote the senescence of immunosuppressive neutrophils in PCa, linking to an unfavorable prognosis 28. The expression and genetic variation of the SLC5A8 gene are closely related to the risk of PCa and its progression 29. PTGS2 plays a crucial role in prostaglandin synthesis, potentially accelerating tumor development and compromising immune response against tumors 30. SLC45A3-ELK4 fusion was found to regulate the proliferation of PCa cells through its non-coding effects 31. Thus far, no research has been reported regarding the roles of PLP1, GSTM4, SLC27A2, PLA2G2C, HMGCLL1 and PLA2G4D in the advancement of PCa.

It is important to validate novel prognostic signature among multiple cohorts. The FAMRGs robustness test was performed in 6 independent PCa cohorts. In each cohort, patients in the low-risk group exhibited a more favorable BCR-free survival rate than those in the high-risk group. Univariate Cox regression analysis indicated that risk scores served as significant predictors of BCR across all cohorts. In TCGA, the AUC values for risk scores predicting 1-, 3-, and 5-year BCR-free survival in PCa patients exceeded 0.95. In all 5 validation cohorts, the AUC values were above 0.6, demonstrating the strong predictive capability of FAMRGs. In addition, FAMRGs showed powerful predictive performance across cohorts compared to 51 previously published PCa prognostic features, apparently superior to other prognostic features. All these findings indicating FAMRGs could serve as a reliable prognostic predictive tool for patients with PCa.

ARSIs, such as abiraterone and enzalutamide, have been widely used in clinical practice, offering survival benefits for patients with advanced PCa, and may become one of the neoadjuvant therapies for high-risk PCa prior to radical prostatectomy 4. Our novel FAMRGs demonstrated an accurate ability to stratify PCa patients for the ARSI treatment response. Regardless of overall survival or progression-free survival, patients with high FAMRGs exhibited an unfavorable prognosis, furnishing evidence to support personalized treatment options for patients.

Considering the unfavorable prognosis observed in patients with high FAMRGs after radical surgery or ARSI treatment, the CTRP drug database was leveraged to identify potential therapeutic agents in the high FAMRGs group, ultimately finding 5 compounds with low IC50 values and high FAMRGs. This finding had important clinical implications for guiding personalized treatment of high FAMRGs patients.

Pathways related to FAMRGs were identified, including E2F targets, G2M checkpoint, DNA replication, mitotic sister chromatid segregation, and mitotic nuclear division, all of which play crucial roles in regulating the tumor cell cycle. Fatty acids are the substrate of sphingolipid synthesis, which plays an important role in cytokinesis 32. The transcriptional regulator of fatty acid synthesis, SREBP1, plays a crucial role in the regulation of cell mitosis 33. Decreased levels of fatty acids can limit the growth of cancer cells 16. It has been reported that inhibiting fatty acid synthesis leads to cell stagnation in the G2M phase, thereby confirming the essential role of de novo lipogenesis in cell cycle completion 34.

The nomogram incorporating clinical factors demonstrated significant clinical utility. Its AUC was superior to any other clinical features. Moreover, the construction of dynamic nomogram enabled us to predict prognosis according to individual characteristics more intuitively and conveniently.

While FAMRGs demonstrated strong performance in forecasting BCR for PCa patients, this study still has some limitations. First of all, the samples in the public database were retrospective, which may exist bias. Secondly, the incomplete clinical information of a small number of samples may lead to the neglect of the potential relationship between FAMRGs and some clinical variables. Finally, the molecular mechanisms of some genes in FAMRGs involved in PCa process have not been clarified, and further experiments are required for validation.

## 5. Conclusion

In conclusion, FAMRGs, the novel prognostic model which is based on machine learning, can accurately classify the risk of PCa patients and is expected to guide the personalized and precise therapeutic strategies for PCa patients.

## Supplementary Material

Supplementary figure and tables.

## Abbreviations

PCa: prostate cancer
FAM: fatty acid metabolism
BCR: biochemical recurrence
ARSI: androgen receptor signaling inhibitor
PSA: prostate specific antigen
RP: radical prostatectomy
NGS: next-generation sequencing
FAs: fatty acids
TCGA: the Cancer Genome Atlas
GEO: Gene Expression Omnibus
MSigDB: Molecular Signatures Database
DEGs: differentially expressed genes
FC: fold change
TMM: Trimmed Mean of M-values
RMA: Robust Multichip Average
K-M: Kaplan-Meier
ROC: receiver opearting characteristic
FAMRGs: FAM-related gene score
Enet: elastic network
GBM: generalized boosted regression modeling
plsRcox: partial least squares regression for Cox
RSF: random survival forest
SuperPC: supervised principal components
suvival-SVM: survival support vector machine
Lasso: least absolute shrinkage and selection operator
HPA: Human Protein Atlas
CTRP: Cancer Therapeutics Response Portal
IC50: half-maximal inhibitory concentration
GSEA: gene set enrichment analysis
GO: gene ontology
HR: hazard ratio

## References

[1] Siegel RL, Giaquinto AN, Jemal A (2024). Cancer statistics, 2024. CA: a cancer journal for clinicians.

[2] Costello AJ (2020). Considering the role of radical prostatectomy in 21st century prostate cancer care. Nature reviews Urology.

[3] Yang J, Xiong X, Liao X, Zheng W, Xu H, Wei Q (2024). Nonsurgical salvage options for locally recurrent prostate cancer after primary definitive radiotherapy: a systematic review and meta-analysis. International journal of surgery (London, England).

[4] Devos G, Devlies W, De Meerleer G, Baldewijns M, Gevaert T, Moris L (2021). Neoadjuvant hormonal therapy before radical prostatectomy in high-risk prostate cancer. Nature reviews Urology.

[5] Van den Broeck T, van den Bergh RCN, Briers E, Cornford P, Cumberbatch M, Tilki D (2020). Biochemical Recurrence in Prostate Cancer: The European Association of Urology Prostate Cancer Guidelines Panel Recommendations. European urology focus.

[6] Falagario UG, Abbadi A, Remmers S, Björnebo L, Bogdanovic D, Martini A (2023). Biochemical Recurrence and Risk of Mortality Following Radiotherapy or Radical Prostatectomy. JAMA network open.

[7] Vale CL, Fisher D, Kneebone A, Parker C, Pearse M, Richaud P (2020). Adjuvant or early salvage radiotherapy for the treatment of localised and locally advanced prostate cancer: a prospectively planned systematic review and meta-analysis of aggregate data. Lancet (London, England).

[8] Teo MY, Rathkopf DE, Kantoff P (2019). Treatment of Advanced Prostate Cancer. Annual review of medicine.

[9] Cornford P, van den Bergh RCN, Briers E, Van den Broeck T, Brunckhorst O, Darraugh J (2024). EAU-EANM-ESTRO-ESUR-ISUP-SIOG Guidelines on Prostate Cancer-2024 Update. Part I: Screening, Diagnosis, and Local Treatment with Curative Intent. European urology.

[10] Mugoni V, Ciani Y, Nardella C, Demichelis F (2022). Circulating RNAs in prostate cancer patients. Cancer letters.

[11] Preisser F, Chun FKH, Pompe RS, Heinze A, Salomon G, Graefen M (2019). Persistent Prostate-Specific Antigen After Radical Prostatectomy and Its Impact on Oncologic Outcomes. European urology.

[12] Van den Broeck T, van den Bergh RCN, Arfi N, Gross T, Moris L, Briers E (2019). Prognostic Value of Biochemical Recurrence Following Treatment with Curative Intent for Prostate Cancer: A Systematic Review. European urology.

[13] Mosele F, Remon J, Mateo J, Westphalen CB, Barlesi F, Lolkema MP (2020). Recommendations for the use of next-generation sequencing (NGS) for patients with metastatic cancers: a report from the ESMO Precision Medicine Working Group. Annals of oncology: official journal of the European Society for Medical Oncology.

[14] Röhrig F, Schulze A (2016). The multifaceted roles of fatty acid synthesis in cancer. Nature reviews Cancer.

[15] Bian X, Liu R, Meng Y, Xing D, Xu D, Lu Z (2021). Lipid metabolism and cancer. The Journal of experimental medicine.

[16] Currie E, Schulze A, Zechner R, Walther TC, Farese RV Jr (2013). Cellular fatty acid metabolism and cancer. Cell metabolism.

[17] Vriens K, Christen S, Parik S, Broekaert D, Yoshinaga K, Talebi A (2019). Evidence for an alternative fatty acid desaturation pathway increasing cancer plasticity. Nature.

[18] Li J, Huang Q, Long X, Zhang J, Huang X, Aa J (2015). CD147 reprograms fatty acid metabolism in hepatocellular carcinoma cells through Akt/mTOR/SREBP1c and P38/PPARα pathways. Journal of hepatology.

[19] Zhou H, Chen Y, Xiao Y, Wu Q, Li H, Li Y (2022). Evaluation of the ability of fatty acid metabolism signature to predict response to neoadjuvant chemoradiotherapy and prognosis of patients with locally advanced rectal cancer. Frontiers in immunology.

[20] Lin L, Yu H, Xie X, Lei Q, Chen X, Su X (2024). Leveraging FAM features to predict the prognosis of LGG patients and immunotherapy outcome. American journal of cancer research.

[21] Shore ND, Moul JW, Pienta KJ, Czernin J, King MT, Freedland SJ (2024). Biochemical recurrence in patients with prostate cancer after primary definitive therapy: treatment based on risk stratification. Prostate cancer and prostatic diseases.

[22] Boehm BE, York ME, Petrovics G, Kohaar I, Chesnut GT (2023). Biomarkers of Aggressive Prostate Cancer at Diagnosis. International journal of molecular sciences.

[23] Xu H, Chen Y, Gu M, Liu C, Chen Q, Zhan M (2021). Fatty Acid Metabolism Reprogramming in Advanced Prostate Cancer. Metabolites.

[24] Wang H, Liu Z, Wang Y, Han D, Du Y, Zhang B (2023). Comprehensive analysis of fatty acid metabolism-related gene signatures for predicting prognosis in patients with prostate cancer. PeerJ.

[25] Tan Z, Deng Y, Cai Z, He H, Tang Z, Feng Y (2024). ACOX2 Serves as a Favorable Indicator Related to Lipid Metabolism and Oxidative Stress for Biochemical Recurrence in Prostate Cancer. Journal of Cancer.

[26] Malek R, Gajula RP, Williams RD, Nghiem B, Simons BW, Nugent K (2017). TWIST1-WDR5-Hottip Regulates Hoxa9 Chromatin to Facilitate Prostate Cancer Metastasis. Cancer research.

[27] Huang H, Li J, Shen J, Lin L, Wu X, Xiang S (2020). Increased ABCC4 Expression Induced by ERRα Leads to Docetaxel Resistance via Efflux of Docetaxel in Prostate Cancer. Frontiers in oncology.

[28] Bancaro N, Calì B, Troiani M, Elia AR, Arzola RA, Attanasio G (2023). Apolipoprotein E induces pathogenic senescent-like myeloid cells in prostate cancer. Cancer cell.

[29] Lin HY, Park HY, Radlein S, Mahajan NP, Sellers TA, Zachariah B (2011). Protein expressions and genetic variations of SLC5A8 in prostate cancer risk and aggressiveness. Urology.

[30] Liu B, Qu L, Yan S (2015). Cyclooxygenase-2 promotes tumor growth and suppresses tumor immunity. Cancer cell international.

[31] Qin F, Zhang Y, Liu J, Li H (2017). SLC45A3-ELK4 functions as a long non-coding chimeric RNA. Cancer letters.

[32] Abe M, Makino A, Hullin-Matsuda F, Kamijo K, Ohno-Iwashita Y, Hanada K (2012). A role for sphingomyelin-rich lipid domains in the accumulation of phosphatidylinositol-4,5-bisphosphate to the cleavage furrow during cytokinesis. Molecular and cellular biology.

[33] Bengoechea-Alonso MT, Ericsson J (2016). The phosphorylation-dependent regulation of nuclear SREBP1 during mitosis links lipid metabolism and cell growth. Cell cycle (Georgetown, Tex).

[34] Scaglia N, Tyekucheva S, Zadra G, Photopoulos C, Loda M (2014). De novo fatty acid synthesis at the mitotic exit is required to complete cellular division. Cell cycle (Georgetown, Tex).

