# Supplementary material for: A Promising Prognostic Signature Consisting of Fatty Acid Metabolism Genes based on Machine Learning Predicts Biochemical Recurrence and Aids ARSI Therapy in Prostate Cancer
Source: J Cancer. 2025 Jul 28;16(11):3450–63. doi: 10.7150/jca.112597 (PMC12374944; doi:10.7150/jca.112597)
Supplement: Supplementary file 1 — Supplementary figure and tables. [file jcav16p3450s1.pdf]

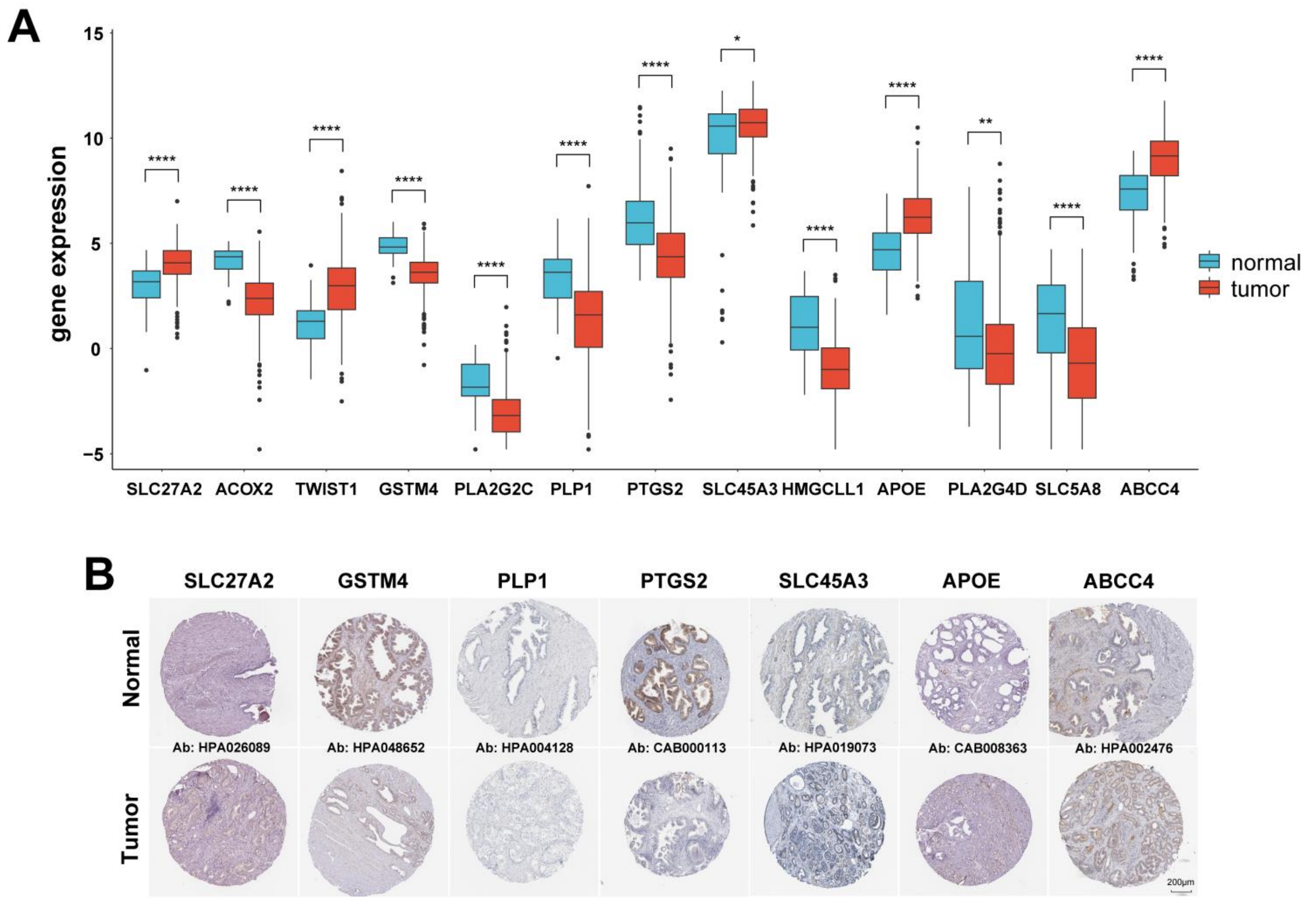

C

Gene	HR	lower 95%CI	upper 95%CI	pvalue
SLC27A2	0.774	0.631	0.950	0.014
ACOX2	0.799	0.698	0.915	0.001
TWIST1	1.226	1.075	1.398	0.002
GSTM4	0.741	0.600	0.915	0.005
PLA2G2C	0.774	0.644	0.930	0.006
PLP1	0.899	0.817	0.988	0.028
PTGS2	0.845	0.755	0.946	0.004
SLC45A3	0.782	0.652	0.938	0.008
HMGCLL1	0.805	0.707	0.916	0.001
APOE	1.370	1.164	1.611	0.000
PLA2G4D	0.886	0.808	0.973	0.011
SLC5A8	0.826	0.755	0.903	0.000
ABCC4	0.852	0.727	0.997	0.046

0.5 1 1.5
Hazard ratios

D

characteristic	HR	lower 95%CI	upper 95%CI	pvalue
SLC27A2	0.825	0.663	1.028	0.086
ACOX2	0.968	0.746	1.256	0.808
TWIST1	1.177	1.034	1.340	0.014
GSTM4	0.844	0.594	1.200	0.345
PLA2G2C	0.864	0.670	1.113	0.257
PLP1	0.968	0.871	1.076	0.551
PTGS2	0.961	0.839	1.100	0.564
SLC45A3	0.978	0.789	1.211	0.836
HMGCLL1	0.928	0.799	1.079	0.331
APOE	1.330	1.106	1.601	0.003
PLA2G4D	0.958	0.867	1.058	0.397
SLC5A8	0.975	0.863	1.101	0.679
ABCC4	0.828	0.682	1.005	0.057

0.5 1 1.5
Hazard ratios

Figure s1. (A) The expression of FAMRGs between normal prostate and prostate cancer tissue. (B) IHC staining of FAMRGs in HPA database. (C) Univariate Cox model of FAMRGs. (D) Multivariate Cox model of FAMRGs.

Table S1. Baseline information of datasets.

Dataset Name	Cancer type	Intervention	Number of Patients
TCGA-PRAD	Prostate cancer	/	463
Cambridge(GSE70768)	Prostate cancer	/	111
Cancer-Map(GSE94767)	Prostate cancer	/	127
CPC-Gene(GSE107299)	Prostate cancer	/	99
DKFZ(EGAS00001002923)	Prostate cancer	/	105
Talor(GSE21034)	Prostate cancer	/	140
PRAD SU2C 2019	Prostate cancer	ARSI	75

Dataset Name	Platform	DOI
TCGA-PRAD	Illumina HiSeq 2000	10.1016/j.cell.2015.10.025
Cambridge(GSE70768)	Illumina HumanHT-12 V4.0 Expression Beadchip	10.1016/j.ebiom.2015.07.017
Cancer-Map(GSE94767)	Affymetrix Human Exon 1.0 ST Array	10.1016/j.euf.2017.01.016
CPC-Gene(GSE107299)	Affymetrix Human Gene 2.0 ST Array (Batch 1,3,4,5), Affymetrix Human Transcriptome Array 2.0 (Batch 2)	10.1016/j.ccell.2019.02.005
DKFZ(EGAS00001002923)	Illumina HiSeq 2000	10.1016/j.ccell.2018.10.016
Talor(GSE21034)	Affymetrix Human Exon 1.0 ST Array	10.1016/j.ccr.2010.05.026
PRAD SU2C 2019	Illumina HiSeq 2000	10.1073/pnas.1902651116

Table S2. URLs that link directly to the information of the images.

Genes	Tissue types	URLs
SLC27A2	Normal	https://www.proteinatlas.org/ENSG00000140284-SLC27A2/tissue/prostate#img
	Tumor	https://www.proteinatlas.org/ENSG00000140284-SLC27A2/cancer/prostate+cancer#img
GSTM4	Normal	https://www.proteinatlas.org/ENSG00000168765-GSTM4/tissue/prostate#img
	Tumor	https://www.proteinatlas.org/ENSG00000168765-GSTM4/cancer/prostate+cancer#img
PLP1	Normal	https://www.proteinatlas.org/ENSG00000123560-PLP1/tissue/prostate#img
	Tumor	https://www.proteinatlas.org/ENSG00000123560-PLP1/cancer/prostate+cancer#img
PTGS2	Normal	https://www.proteinatlas.org/ENSG00000073756-PTGS2/tissue/prostate#img
	Tumor	https://www.proteinatlas.org/ENSG00000073756-PTGS2/cancer/prostate+cancer#img
SLC45A3	Normal	https://www.proteinatlas.org/ENSG00000158715-SLC45A3/tissue/prostate#img
	Tumor	https://www.proteinatlas.org/ENSG00000158715-SLC45A3/cancer/prostate+cancer#img
APOE	Normal	https://www.proteinatlas.org/ENSG00000130203-APOE/tissue/prostate#img
	Tumor	https://www.proteinatlas.org/ENSG00000130203-APOE/cancer/prostate+cancer#img
ABCC4	Normal	https://www.proteinatlas.org/ENSG00000125257-ABCC4/tissue/prostate#img
	Tumor	https://www.proteinatlas.org/ENSG00000125257-ABCC4/cancer/prostate+cancer#img

Table S3. The 117 algorithm combinations of machine learning in this study.

Model	TCGA-PRAD	Cambridge	Cancer-Map	CPC-Gene	DKFZ	Taylor	Average C-index
Lasso + RSF	0.95683977	0.71957672	0.676982592	0.625320161	0.80469124	0.69481401	0.746370749
RSF	0.961485128	0.727513228	0.667864051	0.620929382	0.835806606	0.661960358	0.745926459
StepCox[forward] + RSF	0.961485128	0.727513228	0.667864051	0.620929382	0.835806606	0.661960358	0.745926459
RSF + GBM	0.792588227	0.739417989	0.683890578	0.626051958	0.772618478	0.685853923	0.716736859
StepCox[both] + RSF	0.950461069	0.688492063	0.672008842	0.592023417	0.727142173	0.660602769	0.715121723
StepCox[backward] + RSF	0.950461069	0.688492063	0.672008842	0.592023417	0.727142173	0.660602769	0.715121723
StepCox[forward] + GBM	0.809366983	0.731481481	0.693838077	0.615806806	0.790809	0.645940809	0.714540526
GBM	0.809366983	0.731481481	0.693838077	0.615806806	0.790809	0.645940809	0.714540526
CoxBoost + GBM	0.798204257	0.715608466	0.689416966	0.61909989	0.78602202	0.648112951	0.709410758
Lasso + GBM	0.798204257	0.715608466	0.689416966	0.61909989	0.78602202	0.648112951	0.709410758
StepCox[forward] + survival-SVM	0.671531582	0.738756614	0.662337662	0.605561654	0.815701292	0.659245181	0.692188997
survival - SVM	0.671531582	0.738756614	0.662337662	0.605561654	0.815701292	0.659245181	0.692188997
RSF + Ridge	0.697878389	0.683862434	0.655982316	0.631540432	0.792245093	0.691012761	0.692086904
RSF + survival-SVM	0.691049019	0.685185185	0.650179608	0.623124771	0.80708473	0.691012761	0.691272679
RSF + Enet[α=0.1]	0.701865077	0.67526455	0.659021829	0.63410172	0.781713739	0.687211512	0.689863071
RSF + Enet[α=0.2]	0.702315746	0.674603175	0.65874551	0.630076839	0.781713739	0.687754548	0.689201593
RSF + Enet[α=0.3]	0.703321084	0.67526455	0.65874551	0.629345042	0.779798947	0.686396959	0.688812015
CoxBoost + survival-SVM	0.686784996	0.669973545	0.679745786	0.631174533	0.83245572	0.628292153	0.688071122
Lasso + survival-SVM	0.686784996	0.669973545	0.679745786	0.631174533	0.83245572	0.628292153	0.688071122
StepCox[both] + GBM	0.797684254	0.667328042	0.680298425	0.58507135	0.743417903	0.648384469	0.687030741
StepCox[backward] + GBM	0.797684254	0.667328042	0.680298425	0.58507135	0.743417903	0.648384469	0.687030741
RSF + Enet[α=0.4]	0.703425085	0.668650794	0.655982316	0.626783754	0.779320249	0.68503937	0.686533595
Enet[α=0.1]	0.700859738	0.645502646	0.671732523	0.64032199	0.798468167	0.658702145	0.685931201
StepCox[forward] + Enet[α=0.1]	0.700859738	0.645502646	0.671732523	0.64032199	0.798468167	0.658702145	0.685931201
RSF + Enet[α=0.5]	0.703321084	0.669312169	0.652666482	0.62129528	0.777405457	0.686125441	0.685020986
CoxBoost + Ridge	0.696457048	0.642195767	0.681127383	0.635565313	0.805648636	0.645397774	0.684398653
RSF + Enet[α=0.6]	0.703425085	0.668650794	0.651837524	0.61909989	0.777884155	0.68503937	0.684322803
RSF + plsRcox	0.702905082	0.666666667	0.650179608	0.626417856	0.77453327	0.684496335	0.684199803
RSF + Enet[α=0.7]	0.703667753	0.666666667	0.650179608	0.617636297	0.777884155	0.686125441	0.68369332
Enet[α=0.2]	0.703113083	0.638888889	0.670074606	0.636297109	0.794159885	0.659516698	0.683675045
StepCox[forward] + Enet[α=0.2]	0.703113083	0.638888889	0.670074606	0.636297109	0.794159885	0.659516698	0.683675045
RSF + Enet[α=0.9]	0.703737087	0.666005291	0.650732246	0.618368094	0.775969363	0.686396959	0.68353484
RSF + Enet[α=0.8]	0.703875754	0.665343915	0.651008566	0.617636297	0.776926759	0.686396959	0.683531375
RSF + Lasso	0.70380642	0.66468254	0.651561205	0.618002195	0.775490665	0.685582406	0.683187572
Enet[α=0.3]	0.70436109	0.634259259	0.672008842	0.632638127	0.793681187	0.661960358	0.683151477
StepCox[forward] + Enet[α=0.3]	0.70436109	0.634259259	0.672008842	0.632638127	0.793681187	0.661960358	0.683151477
CoxBoost + Enet[α=0.1]	0.700201068	0.634920635	0.676153634	0.627149652	0.801819052	0.655986967	0.682705168
RSF + CoxBoost	0.703875754	0.661375661	0.653219121	0.616904501	0.77453327	0.686125441	0.682672291
StepCox[forward] + Ridge	0.698641059	0.646164021	0.666482454	0.64032199	0.803255146	0.636166169	0.681838473
Enet[α=0.4]	0.705366429	0.630291005	0.66814037	0.630076839	0.792245093	0.663317947	0.681572947
StepCox[forward] + Enet[α=0.4]	0.705366429	0.630291005	0.66814037	0.630076839	0.792245093	0.663317947	0.681572947
Enet[α=0.6]	0.704846426	0.630952381	0.669521967	0.622758873	0.792245093	0.665761607	0.681014391
StepCox[forward] + Enet[α=0.6]	0.704846426	0.630952381	0.669521967	0.622758873	0.792245093	0.665761607	0.681014391
Enet[α=0.5]	0.706059766	0.628306878	0.669245648	0.627149652	0.792245093	0.66304643	0.681008911
StepCox[forward] + Enet[α=0.5]	0.706059766	0.628306878	0.669245648	0.627149652	0.792245093	0.66304643	0.681008911
RSF + StepCox[forward]	0.701691742	0.662037037	0.646034816	0.618733992	0.773097176	0.682867228	0.680743665
CoxBoost + Enet[α=0.3]	0.70436109	0.628306878	0.67311412	0.623124771	0.794638583	0.660331252	0.680646116
CoxBoost + Enet[α=0.2]	0.702662414	0.628306878	0.671732523	0.62349067	0.797032073	0.658702145	0.680321117
Enet[α=0.7]	0.705054427	0.628968254	0.669521967	0.623124771	0.788894208	0.664947054	0.680085114
StepCox[forward] + Enet[α=0.7]	0.705054427	0.628968254	0.669521967	0.623124771	0.788894208	0.664947054	0.680085114
CoxBoost + Enet[α=0.4]	0.705054427	0.625661376	0.672008842	0.623856568	0.792245093	0.660602769	0.679904846
Enet[α=0.8]	0.704777092	0.627645503	0.666482454	0.620197585	0.790330302	0.665761607	0.67919909
StepCox[forward] + Enet[α=0.8]	0.704777092	0.627645503	0.666482454	0.620197585	0.790330302	0.665761607	0.67919909
CoxBoost + Enet[α=0.5]	0.705435762	0.625	0.671179884	0.62349067	0.790809	0.658973663	0.679148163
StepCox[forward] + CoxBoost	0.704222423	0.635582011	0.660403426	0.614709111	0.790330302	0.669291339	0.679089768
CoxBoost	0.704222423	0.635582011	0.660403426	0.614709111	0.790330302	0.669291339	0.679089768
Lasso + CoxBoost	0.70501976	0.628306878	0.662613982	0.620929382	0.790809	0.664404018	0.678680503
StepCox[forward] + Lasso	0.704742425	0.628306878	0.664548218	0.616538602	0.789372906	0.668205267	0.678619049
Lasso	0.704742425	0.628306878	0.664548218	0.616538602	0.789372906	0.668205267	0.678619049
Enet[α=0.9]	0.704950426	0.625	0.665653495	0.61909989	0.791287697	0.66549009	0.678580267
StepCox[forward] + Enet[α=0.9]	0.704950426	0.625	0.665653495	0.61909989	0.791287697	0.66549009	0.678580267
CoxBoost + Lasso	0.706614435	0.624338624	0.669798287	0.622758873	0.788894208	0.658159109	0.678427256
CoxBoost + Enet[α=0.6]	0.705401095	0.621693122	0.671179884	0.62349067	0.789851604	0.658702145	0.67838642
CoxBoost + Enet[α=0.8]	0.705851765	0.622354497	0.670627245	0.622392975	0.788894208	0.657616074	0.677956127
CoxBoost + Enet[α=0.7]	0.70557443	0.621031746	0.670350926	0.622027076	0.789372906	0.658973663	0.677888458
CoxBoost + Enet[α=0.9]	0.706129099	0.621693122	0.669798287	0.622392975	0.789372906	0.657887592	0.677878997
StepCox[forward] + SuperPC	0.652083478	0.693783069	0.678640508	0.604098061	0.819530876	0.610643497	0.676463248
SuperPC	0.652083478	0.693783069	0.678640508	0.604098061	0.819530876	0.610643497	0.676463248
CoxBoost + StepCox[forward]	0.704777092	0.607804233	0.675600995	0.62129528	0.788894208	0.657616074	0.67599798
Lasso + StepCox[forward]	0.704777092	0.607804233	0.675600995	0.62129528	0.788894208	0.657616074	0.67599798
CoxBoost + plsRcox	0.704603758	0.607804233	0.675600995	0.620929382	0.788894208	0.657344556	0.675862855
Lasso + plsRcox	0.704603758	0.607804233	0.675600995	0.620929382	0.788894208	0.657344556	0.675862855
RSF + SuperPC	0.646363447	0.69047619	0.670074606	0.571899012	0.80708473	0.658702145	0.674100022
RSF + StepCox[both]	0.708798447	0.66005291	0.631666206	0.601170874	0.746290091	0.688840619	0.672803191
RSF + StepCox[backward]	0.708798447	0.66005291	0.631666206	0.601170874	0.746290091	0.688840619	0.672803191
StepCox[both] + survival-SVM	0.709873119	0.617724868	0.6443769	0.61909989	0.75586405	0.660602769	0.667923599
StepCox[backward] + survival-SVM	0.709873119	0.617724868	0.6443769	0.61909989	0.75586405	0.660602769	0.667923599
StepCox[both] + Ridge	0.710150454	0.606481481	0.647692733	0.612513721	0.759693633	0.66549009	0.667003685
StepCox[backward] + Ridge	0.710150454	0.606481481	0.647692733	0.612513721	0.759693633	0.66549009	0.667003685
StepCox[both] + Enet[α=0.1]	0.71053179	0.605820106	0.646034816	0.610318332	0.762087123	0.665761607	0.666758962
StepCox[backward] + Enet[α=0.1]	0.71053179	0.605820106	0.646034816	0.610318332	0.762087123	0.665761607	0.666758962
StepCox[both] + Enet[α=0.2]	0.710115787	0.60515873	0.645482177	0.607391145	0.762087123	0.666304643	0.666089934
StepCox[backward] + Enet[α=0.2]	0.710115787	0.60515873	0.645482177	0.607391145	0.762087123	0.666304643	0.666089934
StepCox[both] + Enet[α=0.3]	0.710150454	0.603835979	0.6443769	0.608122942	0.762087123	0.66549009	0.665677248
StepCox[backward] + Enet[α=0.3]	0.710150454	0.603835979	0.6443769	0.608122942	0.762087123	0.66549009	0.665677248
StepCox[both] + Enet[α=0.4]	0.709630451	0.601851852	0.64410058	0.608122942	0.761608425	0.666033125	0.665224563
StepCox[backward] + Enet[α=0.4]	0.709630451	0.601851852	0.64410058	0.608122942	0.761608425	0.666033125	0.665224563
StepCox[both] + Enet[α=0.6]	0.709699785	0.600529101	0.643271622	0.60848884	0.762087123	0.666576161	0.665108772
StepCox[backward] + Enet[α=0.6]	0.709699785	0.600529101	0.643271622	0.60848884	0.762087123	0.666576161	0.665108772
StepCox[both] + Enet[α=0.5]	0.709838452	0.600529101	0.643547941	0.60848884	0.762087123	0.665761607	0.665042177
StepCox[backward] + Enet[α=0.5]	0.709838452	0.600529101	0.643547941	0.60848884	0.762087123	0.665761607	0.665042177
StepCox[both]	0.709561118	0.599867725	0.64410058	0.608854738	0.761608425	0.666033125	0.665004285
StepCox[backward]	0.709561118	0.599867725	0.64410058	0.608854738	0.761608425	0.666033125	0.665004285
CoxBoost + StepCox[both]	0.709561118	0.599867725	0.64410058	0.608854738	0.761608425	0.666033125	0.665004285
CoxBoost + StepCox[backward]	0.709561118	0.599867725	0.64410058	0.608854738	0.761608425	0.666033125	0.665004285
Lasso + StepCox[both]	0.709561118	0.599867725	0.64410058	0.608854738	0.761608425	0.666033125	0.665004285
Lasso + StepCox[backward]	0.709561118	0.599867725	0.64410058	0.608854738	0.761608425	0.666033125	0.665004285
StepCox[both] + Enet[α=0.8]	0.709665118	0.600529101	0.643271622	0.60848884	0.761608425	0.666304643	0.664977958
StepCox[backward] + Enet[α=0.8]	0.709665118	0.600529101	0.643271622	0.60848884	0.761608425	0.666304643	0.664977958
StepCox[both] + Lasso	0.709734452	0.600529101	0.643824261	0.607757044	0.761608425	0.666304643	0.664959654
StepCox[backward] + Lasso	0.709734452	0.600529101	0.643824261	0.607757044	0.761608425	0.666304643	0.664959654
StepCox[both] + CoxBoost	0.709734452	0.600529101	0.642166344	0.607757044	0.762087123	0.667390714	0.66494413
StepCox[backward] + CoxBoost	0.709734452	0.600529101	0.642166344	0.607757044	0.762087123	0.667390714	0.66494413
StepCox[both] + Enet[α=0.7]	0.709561118	0.600529101	0.643271622	0.608122942	0.762087123	0.665761607	0.664888919

StepCox[backward] + Enet[α=0.7]	0.709561118	0.600529101	0.643271622	0.608122942	0.762087123	0.665761607	0.664888919
StepCox[both] + Enet[α=0.9]	0.709561118	0.600529101	0.642995303	0.607757044	0.761608425	0.666576161	0.664837858
StepCox[backward] + Enet[α=0.9]	0.709561118	0.600529101	0.642995303	0.607757044	0.761608425	0.666576161	0.664837858
StepCox[both] + plsRcox	0.709457117	0.599206349	0.643824261	0.608122942	0.760651029	0.66549009	0.664458631
StepCox[backward] + plsRcox	0.709457117	0.599206349	0.643824261	0.608122942	0.760651029	0.66549009	0.664458631
Ridge	0.716217153	0.570767196	0.655705996	0.628247347	0.77453327	0.543307087	0.648129675
CoxBoost + SuperPC	0.632219372	0.64484127	0.677535231	0.581046469	0.794638583	0.531360304	0.643606871
Lasso + SuperPC	0.632219372	0.64484127	0.677535231	0.581046469	0.794638583	0.531360304	0.643606871
StepCox[forward]	0.718089163	0.553571429	0.64410058	0.611050128	0.764001915	0.502579419	0.632232106
StepCox[forward] + plsRcox	0.718193164	0.548280423	0.6443769	0.615075009	0.765916707	0.497420581	0.631543797
plsRcox	0.718193164	0.548280423	0.6443769	0.615075009	0.765916707	0.497420581	0.631543797
StepCox[both] + SuperPC	0.573008389	0.563492063	0.626416137	0.555067691	0.729535663	0.495519957	0.59050665
StepCox[backward] + SuperPC	0.573008389	0.563492063	0.626416137	0.555067691	0.729535663	0.495519957	0.59050665
